# Cabozantinib and apixaban: an hitherto unreported interaction

**DOI:** 10.1186/s40164-019-0146-9

**Published:** 2019-09-11

**Authors:** Daniele Santini, Fabrizio Citarella, Bruno Vincenzi, Marco Russano, Giuseppe Tonini, Marco Stellato

**Affiliations:** Department of Oncology, Campus Bio-Medico University, via Alvaro del Portillo 200, 00128 Rome, Italy

**Keywords:** Cabozantinib, Metastatic renal cell carcinoma (mRCC), Drug–drug interaction, Apixaban, Cytochrome P450 (CYP 450), P-glycoprotein (P-Gp)

## Abstract

The use of direct oral anticoagulant in cancer patients is an emerging issue, which seems to be an alternative to low molecular weight heparin. Every year several new drugs are approved as anticancer treatment with possible drug-drug interaction with other drugs such as oral anticoagulant. We describe, for the first time, a case of neutropenia and thrombocytopenia in a patient in treatment with cabozantinib, a novel anticancer treatment used in metastatic renal cell carcinoma, and apixaban with promptly resumption of the toxicity after the interruption of cabozantinib. This case suggest a possible interaction between these two pharmaceutical agents, which merit caution considering the spreading of the two drugs.

## To the editor

The management of anticoagulant therapy in cancer patients is an emerging issue because it potentially involves drug–drug interactions (DDI). Recently, last generation direct oral anticoagulants (DOACs) demonstrated to be an available alternative compared to low molecular weight heparin (LMWH) in cancer patients [1]. Two trials published on *The New England Journal of Medicine* demonstrated that apixaban and rivaroxaban reduce the incidence of thromboembolic events in cancer patients. These data stimulated debate about the use of DOACs [2, 3].

Indeed, the use of these drugs in clinical practice is growing and this may lead to possible DDI with several anticancer drugs. We report the case of a man, affected by metastatic renal cell carcinoma (mRCC), intermediate risk score according to International mRCC Database Consortium (IMDC) and chronic kidney disease (CKD, 3b stage). He began treatment with Cabozantinib while he was taking apixaban prescribed 18 months before due to inferior vena cava thrombosis. The patient discontinued cabozantinib administration after 20 days from the start due to a Grade 3 thrombocytopenia (45/ml platelets as nadir) and a Grade 4 neutropenia with a 30/ml neutrophils as nadir.

No other hematological toxicities were reported after changing anticoagulant therapy from apixaban to Nadroparin calcium, with no dose reductions or schedule modifications performed for cabozantinib. Up to date, 2 months after anticoagulant shift, the patient has completed 3 months of cabozantinib therapy and no hematological toxicity was subsequently reported.

Interaction between apixaban and Cabozantinib was suspected.

Cabozantinib is a novel oral multikinase inhibitors including RET, MET, KIT, AXL and VEGFR [4]. METEOR trial demonstrated a longer OS and PFS of this drug compared to everolimus leading to the approval of cabozantinib tables in mRCC and changing, so far, the therapeutic scenario [5].

Interactions between cabozantinib, warfarin and dabigatran [6], but never for apixaban, have been reported. Cabozantinib is mainly eliminated via biliary excretion while urinary excretion is exclusively for its metabolites [6]. Moreover, it is a substrate for Cytochrome P4503A4 (CYP3A4), (with biotransformation in less active metabolites), and a P-glycoprotein (P-gp) inhibitor [6].

About 20% of apixaban undergoes P450 Cytochromes metabolism (mainly via the same CYP3A4), while 30% of the drug is eliminated via urinary excretion. P-gp seems to be involved [7].

We hypothesize that the alterations in cabozantinib pharmacokinetics might have exacerbated the reported hematological toxicity. An excess in free plasma cabozantinib concentration, potentially responsible for hematological toxicity, may be related to a reduction in CYP3A4 bioavailability/activity mainly saturated by circulating apixaban. In addition, CKD was suspected to have been responsible for further apixaban excess and subsequent CYP3A4 saturation. We suppose that these two drugs could interact modifying the pharmacokinetic profile of cabozantinib and amplifying its side effects with the empower of CKD. The clinical scenario described arises the question if this side effect is limited to cabozantinib or could be expectable with other TKI. It is reasonable to assume that this potential pharmacological interaction could be possible for other TKI so that the combination of these different drugs deserve caution. In conclusion, this is the first evidence in literature of a serious hematological toxicity during cabozantinib and apixaban co-administration giving relevance to our report.

## Data Availability

The datasets used and/or analyzed during the current study are available from the corresponding author on reasonable request.

## Abbreviations

DDI: drug–drug interaction
DOACs: direct oral anticoagulants
LMWH: low molecular weight heparin
mRCC: metastatic renal cell carcinoma
CKD: chronic kidney disease
CYP3A4: cytochrome P4503A4
P-gp: P-glycoprotein
